# Evaluation of ceftriaxone utilization in medical and emergency wards of Tikur Anbessa specialized hospital: a prospective cross-sectional study

**DOI:** 10.1186/s40360-016-0057-x

**Published:** 2016-02-18

**Authors:** Alemayehu Sileshi, Admasu Tenna, Mamo Feyissa, Workineh Shibeshi

**Affiliations:** Department of Pharmacy, Arsi University, Asela, Ethiopia; Department of Internal Medicine, School of Medicine, College of Health Sciences, Addis Ababa University, Addis Ababa, Ethiopia; Department of pharmacology and Clinical Pharmacy, School of Pharmacy, College of Health Sciences, Addis Ababa University, Addis Ababa, Ethiopia

**Keywords:** Ceftriaxone, Drug use evaluation, Antibiotic

## Abstract

**Background:**

Ceftriaxone is one of the most commonly used antibiotics due to its high antibacterial potency, wide spectrum of activity and low potential for toxicity. However, the global trend shows misuse of this drug. The aim of this study was to evaluate prospectively the appropriateness of ceftriaxone use in medical and emergency wards of Tikur Anbessa Specialized Hospital.

**Methods:**

A prospective cross-sectional study was conducted by reviewing medication records of patients receiving ceftriaxone during hospitalization at Tikur Anbessa Specialized Hospital between February 1 and June 30, 2014. Drug use evaluation was conducted to determine whether ceftriaxone was being used appropriately based on six criteria namely indication for use, dose, frequency of administration, duration of treatment, drug-drug interaction, culture and sensitivity test. The evaluation was made as per the protocol developed from current treatment guidelines.

**Results:**

The total of 314 records of patients receiving ceftriaxone was reviewed. The prescribing rate of ceftriaxone was found to be very high (58 % point prevalence). Ceftriaxone use was empiric in 274 (87.3 %) cases. The most common indication for ceftriaxone use was pneumonia; observed in 110 (35.0 %) cases. The most common daily dosage, frequency of administration and duration of treatment with ceftriaxone were 2 g (88.9 %), twice-daily (98.4 %) and 8-14 days (46.2 %), respectively. Inappropriate use of ceftriaxone was observed in most of cases (87.9 %), the greatest proportion of which was attributed to inappropriate frequency of administration (80.3 %), followed by absence of culture and sensitivity test (53.2 %).

**Conclusion:**

This study revealed that the inappropriate use of ceftriaxone was very high in the medical and emergency wards of Tikur Anbessa Specialized Hospital. This may lead to emergence of resistant pathogens which in turn lead to treatment failure and increased cost of therapy. Therefore, adherence to current evidence-based guidelines is recommended.

## Background

Ceftriaxone is a broad-spectrum third generation cephalosporin antibiotic for intravenous or intramuscular administration. It is one of the most commonly used antibiotics due to its high antibacterial potency, wide spectrum of activity and low potential for toxicity [1]. The most likely reason for its widespread use is its effectiveness in susceptible organisms in complicated and uncomplicated urinary tract infections, respiratory tract infections, skin, soft tissue, bone and joint infections, bacteremia/septicemia [2], meningitis [3], infections in immunosuppressed patients, acute bacterial otitis media [4], genital infections, disseminated Lyme’s disease and in surgical prophylaxis of infections [5]. It is worthy to note that antimicrobials are among the most commonly used and misused of all drugs [6, 7].

Despite strenuous efforts to control their use and promote optimal prescribing, practitioners still continue to prescribe excessively [8, 9]. But, the inevitable consequence of the widespread use of antimicrobials and extended duration of use, use of suboptimal doses and longer stay in hospitals are additional risk factors that have contributed to the emergence and dissemination of antimicrobial resistance [10]. Antibiotic resistance is a major factor contributing to increased morbidity and mortality of patients as well as cost of medical care. For instance, it is cited in the work of Lee et al. [1] that antimicrobial drug resistance has been projected to add between $100 million and $30 billion annually to health-care costs. In line with this, it was reported that the inappropriate use of ceftriaxone caused, worldwide, an annual cost of $4-5$ million pertaining to infection caused by antibiotic resistant bacteria [11]. The other study conducted in Spain regarding the use of third generation cephalosporins, wherein ceftriaxone was the most frequently prescribed agent, found out that the cost of inappropriate antibiotic use was twice as much for patients who were treated appropriately [12].

The problem of antibiotic resistance has noticeably worsened in Ethiopia during the past several years. Assessment conducted by Food, Medicine and Healthcare Administration and Control Authority of Ethiopia has shown that it is not only higher utilization but also irrational use of antibiotics has been increased. This in turn is also associated with fueling an ever-increasing need for new drugs. Therefore, prudent prescribing of antimicrobial drugs is essential as it may reduce incidences of antimicrobial drug resistance [13, 14].

In Ethiopia, there is no any prospective study that evaluated ceftriaxone utilization. In addition, the retrospective studies conducted regarding this issue were only few. For example, there was retrospective study conducted to comparatively evaluate the use of ceftriaxone in Police Referral Hospital and Tikur Anbessa Specialized Hospital but it involved small sample size [9]. On the other hand, other local retrospective studies done regarding ceftriaxone utilization did not consider duration of treatment and culture and sensitivity test as criteria. Therefore, the present study overcame limitations of the previous studies by using improved study design (prospective cross-sectional study). Furthermore, all wards of the internal medicine and emergency departments were included to enhance the generalizability of the study findings. Thus, the present study is designed to evaluate the appropriateness of ceftriaxone utilization and to assess reasons for its inappropriate use in medical and emergency wards of Tikur Anbessa Specialized Hospital.

## Methods

### Study area description

This study was institution based research conducted in the medical and emergency wards of Tikur Anbessa Specialized Hospital which is located in Addis Ababa, Ethiopia. Tikur Anbessa Specialized Hospital is an 800 bed tertiary care teaching hospital of Addis Ababa University. This hospital offers diagnosis and treatment for approximately 370,000 - 400,000 patients a year.

### Study design

A prospective cross-sectional study was conducted to carry out drug use evaluation by reviewing medical records of patients who received ceftriaxone between February 1 and June 30, 2014. The drug use evaluation was made as per the criteria of the currently developed protocol regarding the rational use of this drug. The treatment protocol was prepared by the joint effort of professionals from School of Pharmacy and School of Medicine. It was prepared by compiling current evidence-based recommendations regarding the use of this drug from WHO guideline 2013, STG of Ethiopia 2010, and other sources of information such as Harrison’s Principles of Internal Medicine 2012, The Sanford Guide to Antimicrobial Therapy 2012, UpToDate, Medscape, and other peer-reviewed journals. More focus was given to “The Sanford Guide to Antimicrobial Therapy” as this guide is among the most widely accepted guidelines in many parts of the world.

### Source and study population

All patients admitted to medical and emergency wards of Tikur Anbessa Specialized Hospital constituted the source population. All in-patients in the medical and emergency wards of Tikur Anbessa Specialized Hospital admitted between February 1 and June 30, 2014 were taken as the study population. All eligible patients included in the study were followed until they complete their treatment with ceftriaxone. To manage patients who transferred from their initial department of admission to another, the mobile number of the patients or their attendants was registered for each patient included in the study (especially for those admitted to the emergency department). Most of them were transferred from the emergency department to internal medicine department, and some were transferred to the orthopedic department.

### Sample size determination

Sample size was calculated using the single proportion formula at 95 % confidence interval and p value of 0.5. The sample size was adjusted based on the total number of patients who were estimated to take ceftriaxone during the study period (N = 923), the required minimum sample with addition of 10 % contingency was finally 299. But, a relatively larger number of participants (314 patients) were included in the present study to maximize its generalizability.

### Inclusion and exclusion criteria

In-patients whose age ≥18 years were eligible provided that they took ceftriaxone during the study period at each of the selected wards. On the other hand, patients who refused to participate in the study and patients with medical records of insufficient or illegible information were excluded. Outpatients were also excluded from the study as it is not convenient to make a follow up study (eg. for any possibility of bleeding and other phenomena).

### Data collection

Data were collected by trained pharmacists via reviewing medication charts of patients admitted during the study period by using patient data collection format. The content of the data collection format was designed to record patient information, disease condition, admission and discharge dates, working diagnosis, past medical history, physical examination, sign and symptoms, abnormal laboratory tests, abnormal diagnostic results, C&S results, information regarding administration of ceftriaxone including its indication, dose, frequency of administration, duration of therapy, and information regarding co-administered medications.

### Key informant interview

Data was collected by self-administered questionnaire to physicians (n = 10) practicing in the infectious disease unit and microbiologists (n = 6) from microbiology laboratory of Tikur Anbessa Specialized Hospital. They were selected based on their long time professional experience in the study area. Accordingly, consultant physicians, senior residents and microbiologists were selected.

### Data quality control

The data collection format was pretested. Additionally, data collectors were trained on how to use such formats and how to approach other health care workers. Furthermore, the data collection process was checked continuously by the principal investigator on daily basis for its completeness and accuracy before the patient gets discharged.

### Data analysis

Drug use evaluation was conducted to determine whether ceftriaxone was being used appropriately based on the protocol currently prepared regarding the rationale use of ceftriaxone. Six criteria namely indication for use, dose, frequency of administration, duration of treatment, drug-drug interaction, culture & sensitivity test were used to evaluate its use.

The data outcomes from those evaluations were entered and analyzed by SPSS version-16.0. In computing the overall appropriateness of ceftriaxone utilization, its use with respect to each of the six criteria was determined for each patient as per the protocol.

The appropriate use of ceftriaxone was computed by dividing the number of cases considered appropriate with respect to all the six criteria to the total number of cases. But, in computing the appropriateness of a given criteria, the number of cases with appropriate dosing was divided by the total number of cases. The responses of key informants were analyzed using content thematic analysis. Accordingly, the collected key informant’s response was first made well familiarized and then significant themes (patterns) were identified. Finally, analysis of the themes were made and contextualized in relation to the existing literature. Binary logistic regression and multivariate logistic regression analysis was made to observe whether there was association between independent variables versus inappropriate ceftriaxone use. Significance of the associations was determined at the p-value of 0.05.

## Results

### Sociodemographic characteristics

A total of 314 patients were included in this study of which 53.8 % were males. Most of the study participants were adults in the age group of 18-65 (90.8 %) with mean age of 37.7 ± 17.2. The socio-demographic characteristics of participants were summarized below (Table 1).

**Table 1:** Socio-demographic characteristics of patients included in the study in medical and emergency wards of Tikur Anbessa Specialized Hospital, 2014 (n = 314)

Characteristics	Category	No (%)
Sex	Male	169 (53.8)
	Female	145 (46.2)
Age	18-65	285 (90.8)
	≥65	29 (9.2)
Department	Internal medicine	231 (73.6)
	Emergency	83 (26.4)
Unit of admission	Non-ICU	294 (93.6)
	ICU	20 (6.4)

*ICU* intensive care unit

### Ceftriaxone prescription pattern

The utilization rate of ceftriaxone was found to be very high (58 % point prevalence) at the medical and emergency wards of Tikur Anbessa Specialized Hospital during the study period. It was found out that 55.1 % of cases received ceftriaxone for diseases where it is indicated as first-line therapy according to current evidence-based guidelines. However, it was prescribed empirically for most of the cases (87.3 %). The top indications for ceftriaxone use were respiratory tract infections (35.4 %), prophylactic indications (11.1 %), and skin, soft tissue and bone infections (10.8 %) (Table 2).

**Table 2:** The prescription pattern of ceftriaxone for the study participants in medical and emergency wards of Tikur Anbessa Specialized Hospital, 2014 (n = 314)

Characteristics	Category		No (%)
Indication of ceftriaxone	Primary		173(55.1)
	Alternative		83(26.4)
	Not indicated		58(18.5)
Type of treatment	Therapeutic	Empiric	274(87.3)
		Specific	5(1.6)
	Prophylactic		35(11.1)
Reasons for ceftriaxone use	Respiratory tract infection		111(35.4)
	Prophylactic indications		35(11.1)
	Skin, soft tissue and bone infection		34(10.8)
	Central nervous system infection		28(8.9)
	Sepsis and septic shock		15(4.8)
	Cardiovascular infection		11(3.5)
	Urinary tract infection		10(3.2)
	Gastro-intestinal infection		6(1.9)
	No indication		58(18.5)

### Dosing and duration of ceftriaxone use

The most commonly prescribed dose of ceftriaxone was 1 g (87.9 %) and most used frequency of administration being twice-daily dosing (98.4 %). The mean duration of treatment was found to be 10.4 days (range: 1-56 days). In most cases, it was used for 8-14 days (46.2 %) (Table 3).

**Table 3:** Dosing and duration of treatment with ceftriaxone in medical and emergency wards of Tikur Anbessa Specialized Hospital, 2014 (n = 314)

Dose (gm)	N (%)	Daily dose (gm)	N (%)	Duration (days)	Frequency (%)
1	276 (87.9)	1	1 (0.3)	1	9(2.9)
1.5	1 (0.3)	2	279 (88.9)	2-7	117(37.3)
2	37 (11.8)	3	1 (0.3)	8-14	145(46.2)
		4	33 (10.5)	15-21	33(10.5)
				>21	10(3.2)

### Culture and sensitivity test

Culture and sensitivity test was not done in most of the patients (89.5 %). In more than half of the patient (53.2 %) this test was not sent for unacceptable reason. Some of the accepted reasons why the test was not sent for investigation were prior initiation of therapeutic antibiotic regimen (25.8 %) and the use of ceftriaxone for its prophylactic indications (10.5 %). Of the 33 cases in which test was done, growth was observed in 8 cases (24.2 %). The organisms were found to be resistant in nearly two third of cases (62.5 %) of cultures on which growth was observed.

### Concomitant administration of drugs

**Fig. 1:**
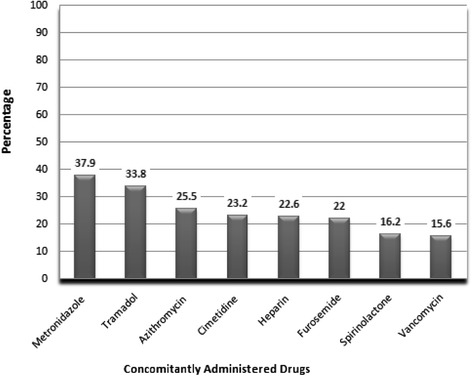
Drugs concomitantly prescribed with ceftriaxone in medical and emergency wards of Tikur Anbessa Specialized Hospital, 2014

As shown in Fig. 1, the most concomitantly administered drugs with ceftriaxone were metronidazole (37.9 %), tramadol (33.8 %), azithromycin (25.5 %) and cimetidine (23.2 %). Inappropriate concomitant use of ceftriaxone was observed as it was co-administered with ringer lactate (observed in 6.7 % of cases). This constituted major drug-drug interaction which may increase the probability of IV incompatibility between the two drugs as a result of binding of ceftriaxone to the calcium contained in ringer lactate. In addition, concomitantly used drugs with moderate interactions were heparin (22.6 %) and warfarin (6.7 %). Six patients (1.9 %) with this type of co-administration experienced either bleeding or increased INR, among which death due to excessive bleeding occurred in one patient.

### Practice of ceftriaxone utilization versus current protocol

**Fig. 2:**
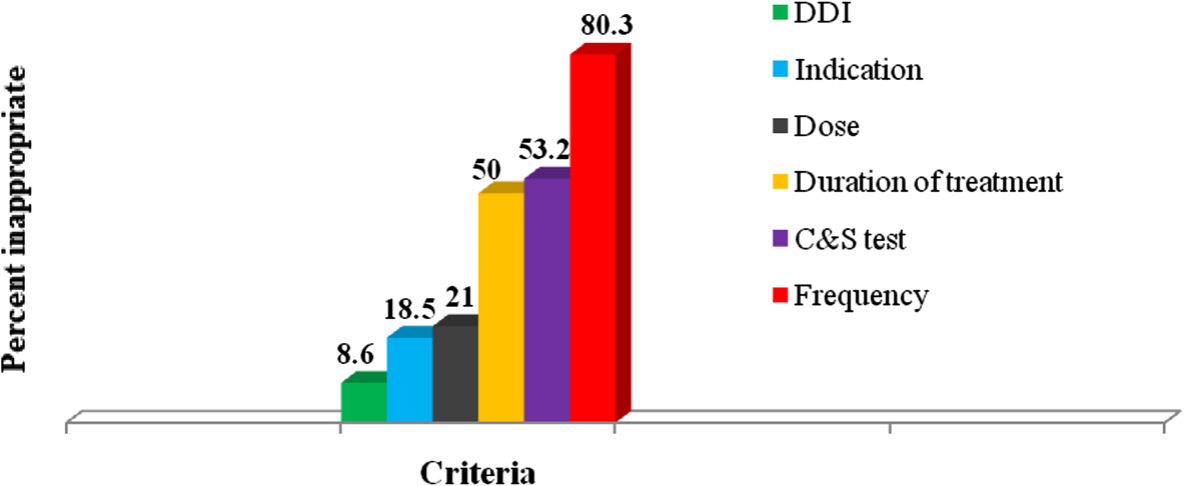
Criteria referenced inappropriate use of ceftriaxone in medical and emergency wards of Tikur Anbessa Specialized Hospital. DDI: drug-drug interaction, C& S: culture and sensitivity test

Most of the ceftriaxone prescriptions (87.9 %) were found to be inappropriate as per the protocol prepared regarding its rationale use. The greatest proportion of inappropriate use was attributed to inappropriate frequency of administration (80.3 %), followed by absence of culture and sensitivity test (53.2 %) and inappropriate duration of treatment (50 %). The remaining inappropriate use was attributed to dose (21 %), indication (18.5 %) and drug-drug interactions (8.7 %) as shown in Fig. 2. Analysis of the practice also indicated that the proportion of inappropriate use was slightly more in the emergency ward compared to the medical wards (90.4 % versus 87 %). In terms of the first few top indications, the analysis of practice indicated that the inappropriate use of ceftriaxone was by far greater than the appropriate use in pneumonia, trauma/injury and wet gangrene (Table 4).

**Table 4:** Appropriateness of ceftriaxone use among the top few indications in medical and emergency wards of Tikur Anbessa Specialized Hospital, 2014 (n = 314)

Indication		Appropriate use N (%)	Inappropriate use N (%)
Pneumonia	CAP	0 (0)	75 (100)
	AP	0 (0)	30 (100)
	HAP	0 (0)	5 (100)
Trauma/Injury		2 (10.5)	17 (89.5)
Pyogenic meningitis		(52.9)	8 (47.1)
Sepsis		9 (69.2)	4 (30.8)
Cellulitis		6 (46.2)	7 (53.8)
Wet gangrene		0 (0)	12 (100)
Brain abscess		7 (63.6)	4 (36.4)

*CAP* community acquired pneumonia, *AP* aspiration pneumonia, *HAP* hospital acquired pneumonia

### Factors associated with inappropriate ceftriaxone use

Analysis using binary logistic regression indicated that gender, age and department type were not significantly associated with inappropriate ceftriaxone use. By contrast, the type of therapy with ceftriaxone was found to have a significant association with inappropriate utilization of this drug (p = 0.002). Accordingly, ceftriaxone use was significantly inappropriate when used as empiric than specific therapy. Multivariate logistic regression analysis was performed to control the effect of any confounder and ensured the presence of such association (Table 5). The responses of key informant interview are summarized in Table 6.

**Table 5:** Factors associated with inappropriate ceftriaxone use at Tikur Anbessa Specialized Hospital

Variable	Appropriateness		COR (95 % CI)	AOR (95 % CI)	*P* value
	No	Yes			
Gender					
Male	147	22	0.829(0.417:1.646)	0.771(0.358:1.658)	0.505
Female	129	16	1.00	1.00	
Age					
18-65	250	35	0.824 (0.237:2.866)	0.669(0.164:2.721)	0.574
>65	26	3	1.00	1.00	
Department					
Emergency	75	8	1.399 (0.614:3.189)	1.557(0.549:4.422	0.405
Internal medicine	201	30	1.00	1.00	
Unit					
Non-ICU	260	34	1.912 (0.604:6.053)	2.535(0.730:8.804)	0.143
ICU	16	4	1.00	1.00	
Treatment type					
Empiric	243	31	31.355(3.395:289.55)	36.98(3.884:352.072)	0.002
Specific	1	4	1.00	1.00	
Diagnosis					
Suspected	146	19	1.123 (0.570:2.213)	1.379(0.648:2.931)	0.404
Confirmed	130	19	1.00	1.00	

*COR* crude odds ratio, *AOR* adjusted odds ratio, *ICU* intensive care unit, *CI* confidence interval

**Table 6:** Responses of the interviewed physicians and microbiologists regarding ceftriaxone use and culture & sensitivity test in medical and emergency wards of Tikur Anbessa Specialized Hospital, 2014

Interview questions	Responses	Number of respondents
Responses of interviewed Physicians		
Why was culture & sensitivity not sent for most of the patients?	Service is not available	8
	Patients come after initiation of antibiotics	5
	Culture results are not reliable	5
	It takes long time to get results back	2
Why was ceftriaxone administered on a twice-daily basis for most cases?	Just because of tradition of practice	5
	There are guidelines which promote it	4
	To ensure its effectiveness	2
Why is the utilization rate of ceftriaxone very high in TASH?	Good availability	8
	Good effectiveness	5
	Low rates of toxicity	4
	Ease of administration	4
Why is ceftriaxone being co-administered with ringers lactate, warfarin and heparin?	Less availability of other drugs	5
	No problem up on such administration	4
	Absence of checking for interaction	2
Why is ceftriaxone used in neutropenic fever,periodontal abscess, etc?	Cost of other more appropriate drugs	4
	Unavailability of other appropriate drugs	4
Why was ceftriaxone used for prolonged duration as in surgical prophylaxis?	Unavailability of equivalent PO medicines	3
	Lack of guidelines	2
	It should not have been used this way	1
Responses of the interviewed microbiologists		
What can you say about the quality of microbiology laboratory?	Poor quality due to the use of expired reagents or antibiotic discs	4
	Currently, its quality is improved	2
Why is most culture & sensitivity tests end up with negative result?	Sample collection after initiation of antibiotics	4
	Use of expired reagents or antibiotic discs	3
	Inappropriate sample collection	2
	Failure to request appropriate laboratory test	1
	Improper use of transporting medium	1
Who will take the bacteriology test result after it is done?	Physicians	5
	Patients	3
	Attendants	1
On average, how long does it take for C&S result to come back (in day)?	Mostly 3 days	5
	Some cultures (eg. blood culture requires 7-14 days)	3

## Discussion

This study was designed to evaluate the appropriateness of ceftriaxone utilization in medical and emergency wards of Tikur Anbesa Specialized Hospital. The current study showed a very high utilization rate of ceftriaxone (58 % point prevalence). This is similar with the results obtained in General Hospital, Port of Spain, in which most of the studied patients (66 %) received ceftriaxone [7]. High rate ceftriaxone prescribing practice was also reported by other studies. This higher utilization of ceftriaxone has been ascribed by the good availability of the drug, good effectiveness and low toxicity rates [15–17].

Ceftriaxone was empirically prescribed (87.3 %) for its therapeutic indication (88.9 %) in our study. This is higher than the empiric use of antibiotics in the study conducted at the University hospital of the West Indies, where two-thirds of patients (67.9 %) were treated with empiric antibiotics [18]. The difference may be attributed to the fact that the latter study included other additional antibiotics in determining the rate of empiric antibiotic use.

In this study, the most common indication for ceftriaxone use was for pneumonia (35 %) followed by respiratory tract infection (35.4 %). This is similar with the finding in Dessie Referral Hospital, wherein the most common indication (36.4 %) of this drug was pneumonia [11]. But in the study conducted at Ayder Referral Hospital, ceftriaxone was most commonly prescribed for preoperative prophylaxis followed by pneumonia [19]. This difference may be attributed to the inclusion of surgery department in the latter study.

It was observed that culture and sensitivity test was not done for more than half of patients (53.2 %) without acceptable reason. This is higher than the result obtained from the study conducted in Korea, in which unacceptable level of culture and sensitivity tests prior to the initial ceftriaxone dose accounted for 33.5 % [1]. In the present study, the interviewed physicians agreed that culture and sensitivity tests were not done in majority of cases and this was ascribed by unavailability of service, unreliable culture result, prior initiation of therapeutic antibiotic regimen and delayed culture result. The interviewed microbiologists agreed that the reason why the bacteriology results were unconvincing could be due to sample collection after initiation of antibiotics, use of expired reagents or antibiotic discs, inappropriate sample collection, improper use of transporting medium, and failure to request appropriate laboratory test. They agreed that the quality of the current microbiology laboratory is poor due to mainly to poor quality reagents and it takes, on average, 3 days for culture results to become available. This is similar with the finding from the study conducted at the University hospital of the West Indies, where culture reports took a mean of 3.7 days to become available [18].

Microbial growth on culture & sensitivity test was observed only in a quarter of samples sent for investigation (24.2 %) in present study. This is lower than the values obtained from studies done at Pakistan (31.4 %), Nepal (47.4 %) and Bangladesh (77 %) [20–22]. The difference may be attributed to the variation in sample sizes and the quality of microbiology laboratory. In the present study, out of cases for which sensitivity was done, resistance to ceftriaxone was seen in nearly two-third of cases (62.5 %). This is similar with the finding from study conducted in Khartoum (64 %) [14]. But it is lower than the finding from study conducted in Bahir Dar (82 %) [23]. This difference could be attributed to differences in the number of tested microorganisms and the prescribing practice of the drug.

In the present study, it was found out that the most common prescribed dose of ceftriaxone was 1 g (87.9 %), whereas, the most common daily dosage was 2 g (88.9 %). This finding is different from the findings from the other similar studies, wherein the most common daily dosage of the drug was 2 g in 63.6 % and 79.4 % cases, respectively [11, 19]. One of the possible reasons for the difference could be the inclusion of patients of all age group in the latter studies in which pediatrics received lower daily doses of ceftriaxone [19].

The other staggering finding in the present study was regarding the frequency of administration with ceftriaxone, where the twice-daily administration accounted for almost all cases (98.4 %). Among the other criteria, frequency of administration took the first place in contributing to the inappropriate use of ceftriaxone; the inappropriate use of ceftriaxone with this criterion was observed in 80.3 % of cases. This is similar with the result obtained in an interventional study done at USA, in which a significant number of patients received a twice-daily dosing of ceftriaxone while they were supposed to receive a once-daily dosing regimen [24]. The reason for administration of ceftriaxone on a twice-daily basis according to key informant physicians interviewed in the present study was just because of tradition of practice.

It was also found out in the present study that the mean duration of treatment with ceftriaxone (10.39 days, range: 1 to 56) is very similar with the findings from the studies conducted at 10 University hospitals of Korea where it was found to be 10.3 days (range, 1 to 61) [1]. But, it is different from the values observed in studies conducted at other hospitals, where it was found to be 7.2 days and 6.8 days, respectively [11, 19]. These differences could be attributed to the difference in patient condition; patients who admit to Tikur Anbessa Specialized Hospital may be those who are, in most cases, terminally-ill requiring longer hospital stay and this in turn may cause physicians to opt long duration of treatment with antibiotics.

The inappropriate duration of treatment with ceftriaxone took the third place in contributing to the overall inappropriate utilization of this drug; observed in 50 % of cases. This was comparable with the finding from the Korean study (42.8 %) [1]. Treatment with ceftriaxone was continued without switching to oral medication in two-third of patients (66.2 %) who deserved switching. Analysis of the practice also indicated that ceftriaxone was used for prolonged duration (4-7 days) in its use for prophylactic purpose as in surgery and trauma. But in these conditions a one-day prophylaxis with the drug is the usual recommendation although up to 3 days may be recommended based on the grade of the wound [25, 26]. The interviewed physicians agreed that such practices were due to lack of guideline and unavailability of equivalent oral medications.

Analysis of the practice indicated that metronidazole took the first place among drugs co-administered with ceftriaxone (37.9 %). Among drugs with potential for interaction, concomitant administration with ringer lactate constituted major drug-drug interaction and was prescribed in a considerable proportion of cases (6.7 %). The most common type of potential drug-drug interaction identified was due to co-administration with heparin (22.6 %) and warfarin (6.7 %). This type of co-administration may result in increased risk of bleeding. In line with this, 6 patients (1.9 %) with this type of co-administration experienced either bleeding or increased INR, among which death due to excessive bleeding occurred in one patient. The interviewed physicians agreed that such practice was due to the less availability of other drugs and absence of checking for possible interaction before prescribing.

The inappropriate use of ceftriaxone was found to be as high as 87.9 % in or study. This finding is similar with the result obtained from the study done at Iran, where the utilization of ceftriaxone was not according to protocol in 85.3 % cases [27]. But, it is higher than the values obtained from studies conducted at Ayder Referral Hospital and Dessie Referral Hospital, in which inappropriate use of this drug was observed in 64.2 % and 46.2 % cases, respectively [11, 19]. These differences may be attributed to the retrospective nature of the studies causing them to consider less number of criteria in evaluating the use of the drug. The other major possible reason for the discrepancy may be attributed to the guidelines used in making the drug use evaluation; the retrospective studies used Ethiopian standard treatment guideline and the present study used current protocol prepared regarding rational use of ceftriaxone.

Analysis using binary logistic regression and multivariate logistic regression indicated that gender, age groups, department types, units of admission and diagnosis types not associated with inappropriate ceftriaxone usage in the present study. This was different from the study done at Thailand, in which female gender was associated with appropriateness of ceftriaxone usage [28]. This difference may be due to the enrollment of more proportion of females in the latter study (60.8 %) compared to the present study (46.2 %). By contrast, multivariate logistic regression showed a significant positive association between empiric treatment and inappropriate ceftriaxone usage in the present study. This implies that empiric treatment with ceftriaxone was significantly associated with its inappropriate use.

## Conclusion

This study revealed that both utilization rate and inappropriate use of ceftriaxone were very high in the medical and emergency wards of Tikur Anbessa Specialized Hospital. This may lead to emergence of resistant pathogens which in turn compromises its effectiveness leading to treatment failure and increased cost of therapy. The inappropriate utilization of ceftriaxone may also compromise patient safety. Therefore, prescribers should limit the use of ceftriaxone only for infections that are proven or strongly suspected to be caused by bacteria. For example, the empiric use of this drug for cases other than its primary indications as in neutopenic fever, lymphadenitis, etc should be avoided. Prescribers should also direct therapy with C&S test result whenever it is possible. Generally, adherence to current evidence-based guidelines is recommended. The hospital (TASH) should also realize continuous and ongoing drug use evaluation; improve the suitability of antibiotics use through the intensification of educational programs, establish an antimicrobial stewardship program, strengthen the DTC unit and capacitate clinical pharmacists in monitoring issues related to drug therapy.

### Ethics committee approval

The confidentiality of data collected from the patients as well as prescribers perspectives, was maintained. As part of this, the identifiers (name and address) of both the patients and prescribers were omitted from the data collection format. Besides, a written consent was obtained for each patient during data collection. Ethical approval was also obtained from the Ethical Review Board of School of Pharmacy and respective departments of School of Medicine, College of Health Sciences, Addis Ababa University.

### Limitations of the study

The present study focused only on internal medicine and emergency departments. But, a more representative result would be obtained if other departments (for example, surgical and orthopedic) were included. Additionally, local prospective studies done on drug utilization of ceftriaxone are limited. Hence, it was not possible to make comparison as the reader wanted to see. The study did not also show the period prevalence of ceftriaxone utilization rather it showed the point prevalence alone.
